# Radiomics of skeletal muscle helps to predict gastrointestinal toxicity in locally advanced rectal cancer patients receiving neoadjuvant chemoradiotherapy

**DOI:** 10.1016/j.ctro.2023.100703

**Published:** 2023-11-20

**Authors:** Wang Yang, Zhiyuan Zhang, Menglong Zhou, Jiazhou Wang, Guichao Li, Yan Wang, Lijun Shen, Hui Zhang, Juefeng Wan, Fan Xia, Zhen Zhang

**Affiliations:** a Department of Radiation Oncology, Fudan University Shanghai Cancer Center, PR China; b Department of Oncology, Shanghai Medical College, Fudan University, Shanghai, PR China

**Keywords:** Rectal cancer, Chemoradiotherapy, Gastrointestinal toxicity, Skeletal muscle index, Body composition, Radiomics

## Abstract

**Background:**

The skeletal muscle index (SMI) can serve as a surrogate for a patient’s nutritional status, which is associated with treatment toxicity. This study aims to investigate the potential of baseline skeletal muscle radiomics features to predict gastrointestinal toxicity of neoadjuvant chemoradiotherapy for rectal cancer.

**Methods:**

A total of 214 rectal cancer patients (115, 49 and 50 in the training, internal and external validation set, respectively) who underwent neoadjuvant pelvic radiotherapy with capecitabine and irinotecan were retrospectively identified. The skeletal muscle at the level of the third lumber vertebra was contoured, and the radiomics features were extracted from computed tomography scans. In the training set, the least absolute shrinkage and selection operator (LASSO) regression algorithm was applied to select features that were most significantly associated with grade 3–4 gastrointestinal toxicity (diarrhea, nausea, vomiting and proctitis). The predictive performance and clinical utility were estimated using the area under the receiver operator characteristic curve (AUC), F1-score and decision curve analysis (DCA).

**Results:**

Nine features, including the SMI and eight radiomics features, were associated with grade 3–4 gastrointestinal toxicity and included in the logistic regression. This combined predictive model, which incorporated the SMI and radiomics features, showed better discrimination than the SMI alone, with an AUC of 0.856 (95 % CI: 0.782–0.929) in the training cohort, 0.812 (95 % CI: 0.667–0.956) in the internal validation cohort and 0.745 (95 % CI: 0.600–0.890) in the external validation cohort. DCA further verified the clinical utility of the combined predictive model.

**Conclusion:**

Radiomics features of skeletal muscle were significantly associated with gastrointestinal toxicity. The predictive model incorporating the SMI and radiomics features exhibits favorable discrimination and may be highly informative for clinical decision-makings.

## Highlights

• The SMI can serve as a surrogate for nutritional status.
• The present study measured skeletal muscle by quantitatively radiomic features.
• A new prediction model incorporating the SMI and radiomics features exhibited favorable discrimination.
• This is the first study to demonstrate the superiority of radiomics features over conventional features of body composition.

## Introduction

Colorectal cancer (CRC) is the third most common malignant cancer and accounts for approximately 9 % of cancer-related deaths worldwide [1]. In the last 20 years, neoadjuvant chemoradiotherapy (CRT) followed by surgical resection has been established as the standard of care in patients with locally advanced rectal cancer (LARC) [2], [3], [4]. However, the optimal choice regarding the concurrent chemotherapy regimen or molecularly targeted drugs during neoadjuvant CRT remains unclear. Several studies with small sample sizes have verified the efficacy of adding irinotecan to neoadjuvant CRT [5], [6]. Recently, neoadjuvant CRT with capecitabine and irinotecan guided by UGT1A1 status was confirmed to significantly improve the pathologic complete response (pCR) rate in a multicenter, randomized phase III trial [7]. Nevertheless, there is concern about the poor tolerability of irinotecan and its propensity to cause neutropenia and diarrhea. Based on previous studies, the incidence of grade 3 or greater gastrointestinal (GI) and hematological toxicity is 11–28 % and 9–31 %, respectively, for the irinotecan regimen [5], [6], [7], [8]. Therefore, there is a great need to identify factors that can precisely predict severe toxicity in rectal cancer patients treated with concurrent CRT.

In addition to disease-specific factors, patient-related factors, such as performance status and nutrition status, are also predictive determinants of toxicity. Several studies have demonstrated that measurement of the skeletal muscle and body composition can serve as a surrogate for a patient’s nutritional status [9]. Characterized by severe loss of skeletal muscle mass and function, sarcopenia is a syndrome associated with worse survival [10], [11], worse tumor response [12], and more postoperative complications [13], [14] in various types of cancer including rectal cancer. Furthermore, low muscle has been shown to be related to dose-limiting toxicities in many different cancer regimens [15], [16], [17]. This is likely in part because chemotherapy dosing were traditionally calculated based on body surface area (BSA), which does not account for variations in body composition. Body composition may be more important in determining the pharmacokinetics of many chemotherapy drugs than BSA. However, there are several limitations of conventional measurements of body composition when applied in predicting treatment outcomes, including ethnic differences in body composition, various definitions and cut-off value for sarcopenia [18], [19] and limited information on conventional imaging data.

Radiomics is an emerging approach that quantitatively and semi-automatically extracts high-dimensional mineable features, including size or volume, shape, intensity and texture [20]. The high-throughput extraction of image features has mainly been applied in oncology to aid in diagnosing cancer [21], predicting the response to treatment [22], [23] and evaluating the prognosis [20]. Moreover, there may be other radiomics features available that describe additional important parameters that may improve prediction of toxicities. With automated imaging methods for body composition available and programs such as radiomics to extract additional features such as texture and shape, this method for predicting toxicity may prove to be quite useful across many cancers and cancer treatments.

Hence, the purpose of this study was to investigate whether baseline skeletal muscle radiomics features can serve as quantitative biomarkers for body composition and help to predict severe gastrointestinal toxicity in patients with LARC treated with neoadjuvant concurrent CRT.

## Materials and methods

### Patent identification

A cohort of 214 patients undergoing neoadjuvant CRT combined with capecitabine and irinotecan was retrospectively identified. Among them, 164 patients enrolled between July 2017 and August 2019 were assigned to the training and internal validation set, while an independent dataset between January 2021 and August 2022 were used for external validation. Patients were eligible if they had histopathologically confirmed rectal adenocarcinoma (i.e., tumors located ≤ 12 cm from the anal verge) and clinical stage T3-4 and/or N + disease, as assessed on magnetic resonance imaging (MRI). Patients with T2N0M0 (stage I) disease who had strong desire to preserve the rectum and those with resectable oligometastasis (stage IV) were also permitted to enroll. Axial computed tomography (CT) images at the level of the third lumbar vertebra (L3) were obtained prior to treatment in all cases. The cohort was first randomly divided into a training set and a internal validation set based on a 7:3 ratio.

### Treatment delivery and toxicity

All enrolled patients received neoadjuvant CRT delivered with a 6-MV linear accelerator using intensity-modulated radiation therapy (IMRT). The radiation dose was 50 Gy with a daily fraction of 2.0 Gy. The clinical target volume (CTV) encompassed all macroscopic disease (rectal and nodal) as well as the internal iliac lymph nodes and the mesorectum (*peri*-rectal fat and the presacral space). The planning target volume (PTV) consisted of a 5–8 mm expansion around the CTV considering the individual uncertainties. Image-guided radiotherapy procedure was routinely performed using once weekly cone-beam computed tomography (CBCT) and two-dimensional (2D) imaging over the treatment duration. The Dmax of the small bowel was ≤ 50 Gy, the V45 of the small bowel was ≤ 100 cc, the Dmean of the femoral heads were ≤ 30 Gy, the V45 of the bladder was ≤ 50 %. Concurrent chemotherapy regimens included capecitabine 625 mg/m^2^ twice daily 5 d/wk and irinotecan guided by uridine diphosphate glucuronosyltransferase 1A1 (UGT1A1) status. The UGT1A1*28 confers reduced UGT1A1-mediated inactivation of SN-38, which has been considered as a major predictive marker for adverse effects [24]. Irinotecan was intravenously administered at a weekly dose of 80 mg/m^2^ among patients with the UGT1A1*28 6/6 genotype and 65 mg/m^2^ among those with the UGT1A1*28 6/7 genotype. The use of preoperative or postoperative chemotherapy regimens, including oxaliplatin, fluorouracil and leucovorin (FOLFOX), capecitabine and oxaliplatin (XELOX) or irinotecanand capecitabine (XELIRI), was decided by each physician.

Toxicity data were recorded according to the National Cancer Institute’s Common Terminology Criteria for Adverse Effects (CTCAE), version 5.0[25]. Acute toxicity was defined as toxicity occurred during radiotherapy and in the first 3 months thereafter. As GI toxicities, diarrhea, nausea, vomiting and proctitis were assessed. Each of our patients was evaluated by the physicians, and data were recorded in a standardized form placed in the medical file weekly throughout the treatment course and monthly up to 3 months from the end of radiation. Data on GI toxicities were determined based on the highest recorded toxicity grade.

### Image acquisition and radiomics feature extraction

**Fig. 1:**
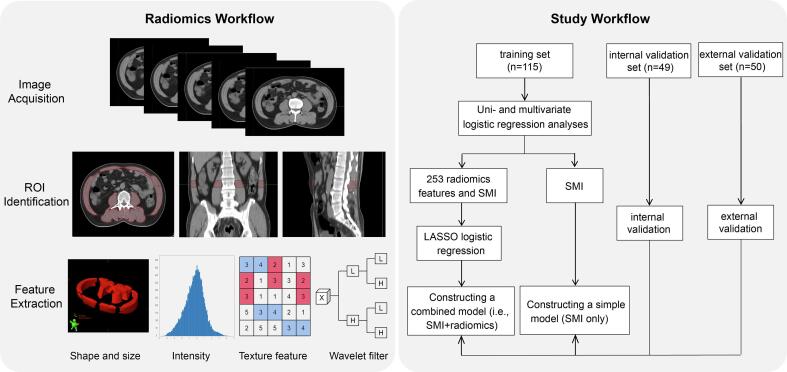
Schematic of the workflow. Abbreviations: ROI, region of interest; LASSO, least absolute shrinkage and selection operator; SMI, skeletal muscle index.

The radiomics workflow and study flowchart are shown in Fig. 1. For all patients, the body composition and radiomics features of skeletal muscle were assessed on the CT scan at baseline. CT examinations were conducted using a multi-detector CT scanner（Somatom Definition, Siemens, Erlangern, Germany or PHILIPS Brilliance Big Bore, Philips Healthcare, Cleveland, OH）with non-contrast enhancement. The CT protocols were conducted according to the following parameters: tube voltage: 120 kV, tube current: 160–261 mA, matrix: 512 × 512, helical pitch: 1 and slice thickness: 3–5 mm. To minimize potential bias arising from variations in muscle distribution within a single slice and facilitate the integration of 3D volumetric data into the radiomic analysis, five consecutive transverse slices at the level of the third lumbar vertebra (L3) were contoured to measure the cross-sectional area and radiomics features of skeletal muscle with MIM (version 7.0; MIM Software, Inc., Cleveland, OH), as previously described [26]. Skeletal muscle was identified based on a pre-established threshold range of −29 to + 150 HU, and boundaries were corrected manually when necessary. The cross-sectional area of skeletal muscle was further normalized to the patient’s height (m^2^), resulting in the skeletal muscle index (SMI, cm^2^/m^2^).

After image segmentation, a total of 253 radiomics features were extracted from the regions of interest (ROI) on the images using an in-house algorithm in MATLAB (MathWorks, Inc., Natick, MA). Extracted radiaomics features were listed in Supplementary Table E1. The radiomics features included shape and size-based features, intensity features, texture features and wavelet features. After feature extraction, the data was pre-processed using the preProcess function, which involved Box-Cox transformation, centering, and scaling to normalize the data.

### Radiomics feature selection, model construction and statistical analysis

The SMI and 253 radiomics features were all included for feature selection to build a combined model. The least absolute shrinkage and selection operator (LASSO) regression algorithm, which is widely used for the dimensionality reduction of high-dimensional data [27], was applied in the training cohort to select gastrointestinal toxicity-related features. Using the radiomics score (rad-score), which was a risk score calculated as a linear combination of the selected features weighted by their respective coefficients, a combination of the SMI and selected radiomics features was constructed to reflect the probability of gastrointestinal toxicity for each patient.

K-fold cross-validation (k = 5) was applied to evaluate the model' s performance on multiple subsets of the data. K-fold cross-validation is one of the widely used methods in which the data are split into k subsets. The model is then trained and evaluated k times. In each iteration of the k-fold process, one subset serves as the validation set, while the remaining k-1 folds subsets are employed for training purpose [28].

The concordance index (C-index) for the models, the value of which equals the area under the receiver operating characteristic (ROC) curve (AUC), was used to depict the discrimination of the model in the training cohort and then validated in the validation cohort. We calculated the F1 score as an additional evaluation metric, alongside the AUC, to assess the model’s performance, especially in case of imbalanced data learning. Decision curve analysis (DCA) was also applied to determine the clinical utility of the prediction model by quantifying the net benefits at different threshold probabilities in the training and validation cohorts [29]. This methodology is a tool for assessing and comparing prediction models, molecular markers, and diagnostic tests [30], [31], [32]. Importantly, evaluating net benefit is recommended by the TRIPOD guidelines for prediction models [33].

All statistical tests were performed using the R statistical software package (version 3.6.0; R Foundation for Statistical Computing, Vienna, Austria). The “caret”, “dplyr” and “glmnet” packages were used for data analysis and model construction. The “pROC”, “rmda” and “ggplot2” packages were used for ROC curve plotting, DCA and result visualization. Intergroup comparisons were analyzed in the training and validation set using the Student *t* test or Mann-Whitney *U* test for continuous variables, and chi-squared test or Fisher exact tests for categorical variables, as appropriate. A two-sided P-value < 0.05 was considered statistically significant.

## Results

### Patients characteristics

In total, 214 patients were included in the study. Among them, 115 patients were distributed to the training set, and 49 patients were distributed to the validation set. An independent dataset comprising 50 patients were used for external validation. The baseline demographics, clinicopathological characteristics and treatment toxicity in the training and validation cohorts are summarized in Table 1. No significant difference was observed between the training, internal and external validation datasets. Overall, the mean ± SD of the BMI and SMI was 23.2 ± 2.8 kg/m^2^ and 46.8 ± 7.5 cm^2^/m^2^, respectively, at baseline. All patients received radiotherapy combined with concurrent capecitabine and weekly irinotecan, and 77.1 % of patients received 4–5 cycles of concurrent irinotecan. Table E2 illustrates the incidence and maximum toxicity observed during concurrent chemoradiotherapy in detail. The incidence of grade 3 or greater gastrointestinal and hematologic toxicity in the current study was 29.9 % and 30.8 %, respectively. The most severe GI toxicity occurred in the fourth to fifth week.

**Table 1:** Baseline demographics, clinicopathological characteristics and treatment toxicity in the training, internal and external validation cohorts.

Characteristics	Training cohort (n = 115)	Internal validation cohort (n = 49)	External validation cohort (n = 50)	P value
Sex				0.542
Male	82(71.3 %)	31(63.3 %)	36(72.0 %)	
Female	33(28.7 %)	18(36.7 %)	14(28.0)	
Age (years)	55.4 ± 10.3	54.5 ± 9.9	51.5 ± 8.3	0.063
Height (cm)	166.5 ± 7.8	167.7 ± 8.5	166.5 ± 7.3	0.664
Weight (kg)	64.1 ± 9.4	65.7 ± 10.0	65.1 ± 10.5	0.605
BMI (kg/m^2^)	23.1 ± 2.7	23.3 ± 2.6	23.4 ± 3.2	0.721
SMI (cm^2^/m^2^)	47.2 ± 7.5	44.9 ± 6.3	47.7 ± 8.4	0.119
cTNM stage				0.718
I	2(1.8 %)	2(4.1 %)	1（2.0 %）	
II	6(5.3 %)	2(4.1)	5（10.2 %）	
III	94(81.7 %)	39(79.6 %)	40（81.6 %）	
IV	11(9.6 %)	6(12.2 %)	3（6.1 %）	
Unknown	2(1.7 %)	0(0.0 %)	0（0.0 %）	
ypTNM stage				0.778
0(pCR)	14(12.2 %)	9(18.4 %)	10(20.0 %)	
I	19(16.5 %)	7(14.3 %)	10(20.0 %)	
II	19(16.5 %)	9(18.4 %)	10(20.0 %)	
III	32(27.8 %)	9(18.4 %)	9(18.0 %)	
IV	8(7.0 %)	6(12.2 %)	3(6.0 %)	
Unknown	23(20.0 %)	9(18.4 %)	8(16.0 %)	
TRG				
0	20(17.4 %)	9(18.4 %)	10(20.0 %)	0.997
1	19(16.5 %)	9(18.4 %)	10(20.0 %	
2	42(36.5 %)	17(34.7 %)	18(36.0 %)	
3	11(9.6 %)	5(10.2 %)	3(6.0 %)	
Unknowm	23(20.0 %)	9(18.4 %)	9(18.0 %)	
Weekly irinotecan cycles				
1–3	34(29.6 %)	8(16.3 %)	7(14.0 %)	0.042
4–5	81(70.4 %)	41(83.7 %)	43(86.0 %)	
Grade 3/4 overall toxicity	55(47.8 %)	23(46.9 %)	28(56.0 %)	0.576
Grade 3/4 gastrointestinal toxicity	30(26.1 %)	17(34.7 %)	17(34.0 %)	0.420
Grade 3/4 hematologic toxicity	39(33.9 %)	11(22.4 %)	16(32.0 %)	0.340

Abbreviations: BMI, body mass index; SMI, skeletal muscle index; pCR, pathologic complete response; TRG, tumor regression grading.

### Features selection and model construction

According to the uni- and multivariate logistic regression analyses, the rad-score was identified as the only independent predictor of grade 3/4 gastrointestinal toxicity (Table 2). Given that the SMI is considered a strong predictor of treatment toxicity based on previous studies [16], [17], it was also included in model construction. The variance inflation factor (VIF) was 2.434, indicating no collinearity between the two variables in the collinearity diagnosis. In total, 253 radiomics features, as well as the SMI, were extracted from L3 skeletal muscle on the pretreatment CT scans. Nine features with nonzero coefficients were selected in the training set using LASSO logistic regression analysis, which can be applied for biomarker selection in high-dimensional data (Fig E1A and E1B). Selected features with their coefficient values are listed in Table E3.

**Table 2:** Uni- and multivariate logistic regression analyses of predictive factors associated with grade 3/4 gastrointestinal toxicity.

Characteristics	Univariate analyses		Multivariate analyses	
	OR(95 % CI)	P value	OR(95 % CI)	P value
Sex (Male vs. Female)	0.458(0.188–1.112)	0.084	–	–
Age (years)	1.020(0.977–1.065)	0.358	–	–
Height (cm)	1.025(0.969–1.083)	0.388	–	–
Weight (kg)	0.965(0.921–1.011)	0.134	–	–
BMI (kg/m^2^)	0.811(0.685–0.960)	**0.015**	0.957(0.768–1.193)	0.698
Weekly irinotecan cycles (1–3 vs. 4–5)	3.080(1.273–7.452)	**0.013**	2.872(0.910–9.066)	0.072
cTNM stage			–	–
I	reference			
II	4.000(0.167–95.756)	0.392		
III	2.000(0.201–19.914)	0.554		
IV	1.278(0.253–6.451)	0.767		
ypTNM stage			–	–
0	reference			
I	1.167(0.089–15.321)	0.907		
II	2.187(0.214–22.337)	0.509		
III	4.083(0.412–40.455)	0.229		
IV	2.333(0.248–21.981)	0.459		
TRG	1.350(0.803–2.269)	0.258	–	–
pCR (0 vs. 1)	2.034(0.419–9.880)	0.379	–	–
SMI (cm^2^/m^2^)	0.878(0.819–0.941)	**<0.001**	1.094(0.974–1.230)	0.129
Radiomics score (per 0.1)	3.372(2.010–5.656)	**<0.001**	4.537(2.194–9.381)	**<0.001**

Abbreviations: OR, odds ratio; CI, confidence interval; BMI, body mass index; TRG, tumor regression grading; pCR, pathologic complete response; SMI, skeletal muscle index;

The P < 0.05 was considered statistically significant and was represented as bold.

Although SMI was found to be related with hematologic toxicities, the model we constructed for predicting hematologic toxicities demonstrated suboptimal discriminatory ability. Incorporating radiomics features and constructing more complex models did not improve the model’s performance.

### Model performance in the training, internal and external validation sets

**Fig. 2:**
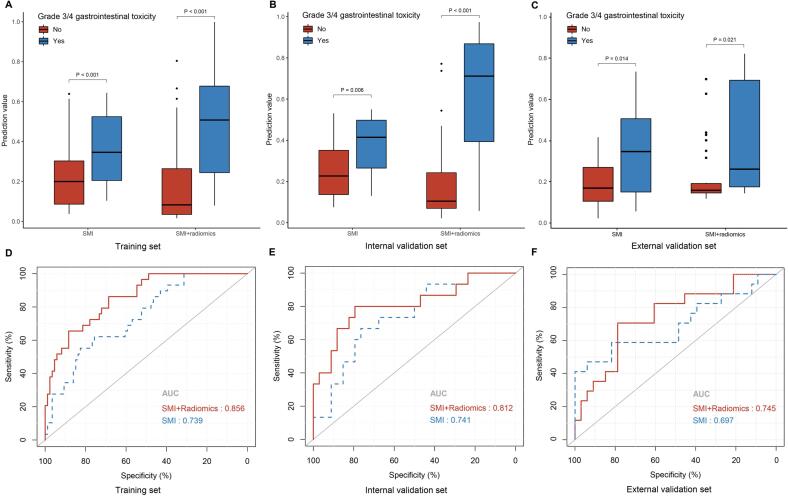
Boxplots of the predictive value in the training (A) internal validation (B) and external validation (C) sets. The blue bars represent patients who developed grade 3/4 gastrointestinal toxicity, whereas the red bars represent patients who did not. The predictive performance of the SMI model and the combined model (i.e., SMI + radiomics) for predicting grade 3/4 gastrointestinal toxicity, as presented by ROC curves, in the training (D), internal validation (E) and external validation (F) sets. Abbreviations: SMI, skeletal muscle index; ROC, receiver operator characteristic.

A significant difference in the predictive value was noted between patients who develop grade 3/4 gastrointestinal toxicity and those who did not according to the predictive models constructed by the SMI alone and the SMI + radiomics features in the training set (0.200 vs. 0.346, P < 0.001; 0.083 vs. 0.508, P < 0.001, respectively, Fig. 2A); this difference was also verified in the internal and external validation set (internal validation set: 0.227 vs. 0.415, P = 0.006; 0.105 vs. 0.712, P < 0.001, external validation set: 0.193 vs. 0.355, P = 0.014; 0.186 vs. 0.342, P = 0.021, Fig. 2B and 2C).

ROC curves comparing the discriminatory efficiency of the SMI and the combined model (i.e., SMI + radiomics) in the training, internal and external validation sets are shown in Fig. 2D-2F. Both models showed favorable discrimination, with an AUC of 0.739 (95 % CI: 0.639–0.839) and 0.856 (95 % CI: 0.782–0.929), respectively, in the training set, which was validated in the validation sets, with an AUC of 0.741 (95 % CI: 0.594–0.889) and 0.812 (95 % CI: 0.667–0.956) in the internal validation set and 0.697 (95 % CI: 0.526–0.867) and 0.745 (95 % CI: 0.600–0.890) in the external validation set, respectively. The F1-score in the training set demonstrated improvement from 0.553 in the SMI model to 0.617 in the combined model, with a consistent trend observed in both the internal (0.606 to 0.706) and external validation sets (0.584 to 0.667). The combined model, constructed by the SMI and radiomics features, showed better predictive efficacy than the model based on the SMI alone. The sensitivity, specificity, accuracy, precision and F1-score of the two models are listed in detail in Table E4.

**Fig. 3:**
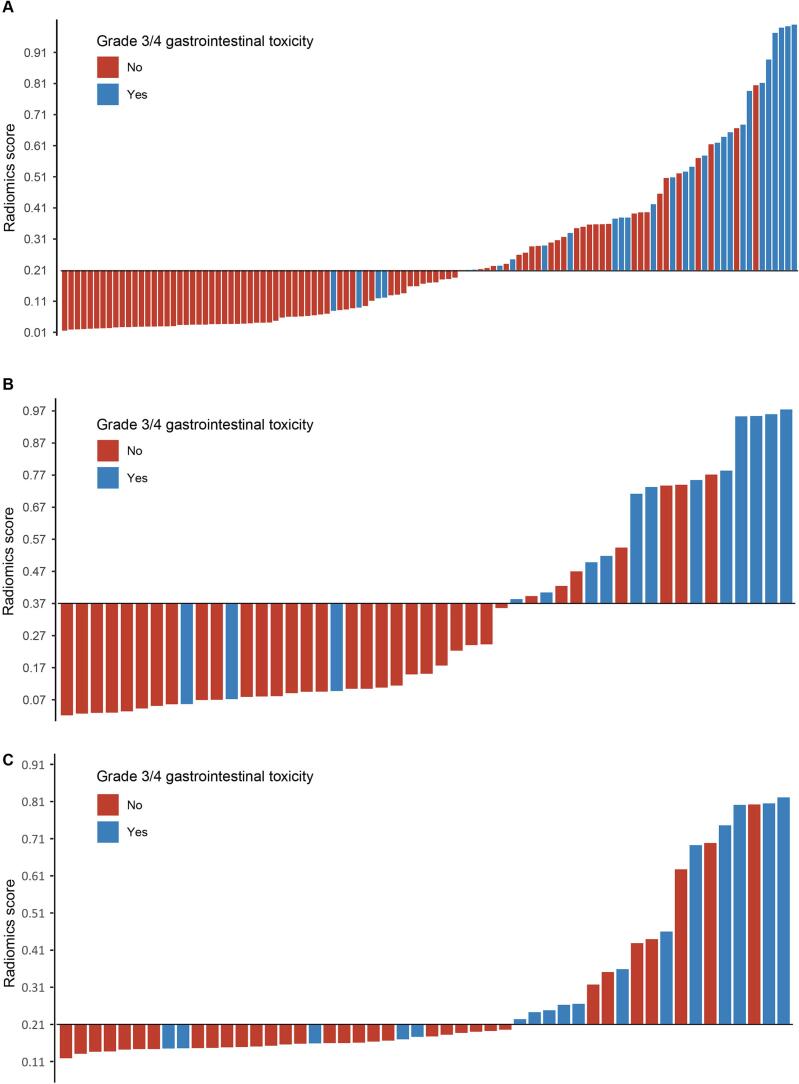
Waterfall plot for the distribution of the radiomics score and grade 3/4 gastrointestinal toxicity based on the combined model in the training (A), internal validation (B) and external validation (C) sets.

The waterfall plot depicted the distribution of the rad-score based on the combined model. The dividing line was drawn at the optimal cutoff value, which was calculated by the Youden index in the training, internal and external validation datasets (Fig. 3).

### DCA

**Fig. 4:**
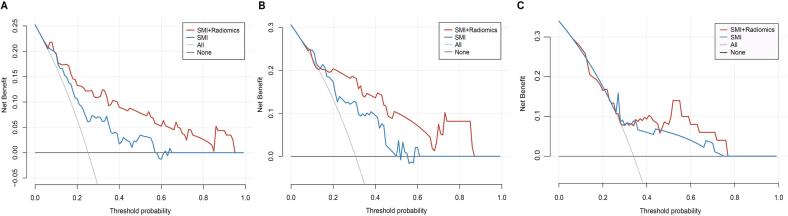
DCA of the two predictive models in the training (A), internal validation (B) and external validation (C) sets. The y-axis indicates the net benefit; the x-axis represents the threshold probability. The red line represents the predictive model constructed by the SMI and radiomics features. The blue line represents the SMI model. The black line represents the hypothesis that no patients developed grade 3/4 gastrointestinal toxicity. The grey line represents the hypothesis that all patients developed toxicity. The decision curves reveal that applying the combined model adds more benefit than the SMI model if the threshold probability of grade 3/4 gastrointestinal toxicity ranges from 0.10 to 0.94 in the training set, 0.17 to 0.86 in the internal validation set and 0.35 to 0.76 in the external validation set, respectively. Abbreviation: DCA, decision curve analysis; SMI, skeletal muscle index.

The decision curves are shown in Fig. 4 for the training, internal and external validation cohorts. Both models showed relatively good performance, with a greater net benefit than either the treat-all or treat-none scheme for a wide range of threshold probabilities. When the threshold probability of grade 3/4 gastrointestinal toxicity was between 0.10 and 0.94 in the training set, between 0.17 and 0.86 in the internal validation set and between 0.35 and 0.76, applying the combined model added more benefit than applying the SMI model alone.

## Discussion

Although the efficacy of concurrent irinotecan has been confirmed by prospective randomized clinical trials [7], gastrointestinal toxicity seems to be the most important obstacle to its use. Thus, an improved method for predicting toxicity is urgently needed. The present study developed and validated a CT-based combined model incorporating the SMI and radiomics features for predicting grade 3/4 gastrointestinal toxicity in patients with LARC undergoing neoadjuvant concurrent CRT and exhibited favorable discrimination, with a C-index of 0.856 in the training set, 0.812 in the internal validation set and 0.745 in the external validation set. This is the first exploratory study to predict severe treatment toxicity by applying skeletal muscle radiomics features and the first to demonstrate the superiority of radiomics features over conventional measurements of body composition.

Body composition has been verified as a determinant of treatment toxicity in prior work. Indeed, Prado et al.[34] revealed that a low lean body mass was a predictor of toxicity in female patients administered fluorouracil (5-FU) and proposed that variation in toxicity between males and females may be partially explained by body composition. Furthermore, as the most widely used characteristic of body composition, the SMI is significantly associated with severe toxicity in various malignancies, such as esophagogastric cancer [35], colorectal cancer [17], breast cancer [36], and renal cancer [37], resulting in the termination of treatment and a worse prognosis. Consistent with preceding studies, the SMI was closely related to grade 3/4 gastrointestinal toxicity in patients undergoing neoadjuvant CRT in this study. A simple model constructed based on the SMI alone showed acceptable performance with a C-index of 0.739, 0.741 and 0.697 in the training, internal and external validation cohorts, respectively, but the sensitivity was unsatisfactory at only 55.2 %. Thus, the SMI may serve as a simple and feasible tool for predicting toxicity, but there is a critical need for a better predictive model.

Radiomics is a novel high-throughput method for extracting important high-dimensional data from medical images that has shown great potential in diagnosis and prognosis prediction. In terms of toxicity prediction, many radiomics-based models have been found to predict treatment-related toxicity. Bousabarah et al.[38] investigated 110 non-small-cell lung cancer (NSCLC) patients treated with stereotactic body radiation therapy (SBRT) and concluded that CT-based radiomics analysis of the primary tumor may help to predict the development of local lung fibrosis. Abdollahi et al.[39] extracted radiomics features from pre-/post-radiation rectal wall MR images of prostate cancer patients to predict rectal toxicity. The pre-radiation T2-weighted and apparent diffusion coefficient (ADC) radiomics models yielded an AUC of 0.68 and 0.58, respectively, and the AUC was 0.81 when all features were included.

Nevertheless, few studies have evaluated body composition with radiomics analysis. The combination of radiomics and skeletal muscle has only been attempted by Jong [40] in a post hoc analysis of a randomized clinical trial including 116 patients with stage IV NSCLC. The author aimed to demonstrate the association of skeletal muscle radiomics features and future muscle loss but showed poor discrimination of the baseline radiomics model, with an AUC of 0.49 (95 % CI: 0.36–0.62). Nevertheless, the rad-score was identified as the only independent predictor for gastrointestinal toxicity in the current study, and a combined model consisting of radiomics features and the SMI showed excellent discrimination. Compared with the model based on the SMI alone, this combined model increased the C-index from 0.739 to 0.856, 0.741 to 0.812 and 0.697 to 0.745 in the training, internal and external validation set, respectively. Notably, the sensitivity of the model significantly improved from 55.2 % to 86.2 % in the training cohort, and yielded similar improvement in the internal and external validation cohort. Moreover, the combined model added more benefit than the SMI model over a wide range of threshold probabilities, suggesting that the radiomics analysis of skeletal muscle may be a more accurate and sensitive predictor for treatment-related toxicity. Although the primary endpoint of these two studies may vary, the large difference in model performance between the current study and that reported by Jong [40] may be partially attributed to the overall thickness of the ROI. Indeed, radiomics extraction of high-dimensional features relies on a three-dimensional morphology, suggesting that including more slices containing high-order features may be preferable.

Apart from SMI, eight radiomics features were indentified in the model, including four features correlated with texture and four with intensity. Most of the selected features was transformed by wavelets, which was a time scale methods act as a “mathematical microscope” through which one can evaluate different part of the signal by adjusting the focus [41]. The most distinct radiomics features was features in the Grey Level Co-occurrence Matrix (GLCM) and Grey Level Run Length Matrix (GLRLM) category (i.e., Wavelet(LL)_GLCM_Correlation, Wavelet(LL)_GLRLM_SRLGE and Wavelet(HL)_GLRLM_SRE). They described the element similarity inside GLCM, the joint distribution of short run length with low grey-level values, and distribution of short run length after wavelet filter transformation, respectively, which all of them reflect textual heterogeneity within the skeletal muscle. The rationale for the connection of radiomics and skeletal muscle was that patients with muscle depletion had a tendency to develop deposition of inter-muscular adipose tissue, leading to the heterogeneity of intensity and texture. The other four features derived from intensity also yielded significant information, and this may be attributed to the similar reason.

The current study strictly enrolled patients who received concurrent capecitabine and irinotecan to avoid the bias caused by various chemotherapy regimens. Although the model was derived by specific treatment, we develop a novel method incorporating conventional parameters and radiomics features of body composition to predict toxicity, which may be theoretically widespread applied to study other areas such as other chemotherapy regimens, target therapy and immunotherapy.

There are several limitations to the present study, one of which is the retrospective design, which may have introduced selection bias. Second, the model was trained and validated at one institution. Multi-institution validation with a larger sample of patients is required to further demonstrate the robustness of our findings. Third, molecular characteristics, such as the UGT1A1 genotype or status of other genes, have not yet been incorporated into the predictive model. Fourth, image segmentation was performed manually, which is time-consuming work that weakens the clinical application of this method in large cohorts; however, this restriction can be solved by the use of autosegmentation software that has been developed in recent years [42]. Finally, incorporating radiomics features and constructing more complex models did not enhance the model’s performance for predicting hematologic toxicities. Further investigation is warranted, potentially involving the refinement of methodology, the addition of relevant factors, or the exploration of advanced modeling techniques to yield more definitive evidence.

## Conclusion

The current study illustrates that baseline skeletal muscle radiomics features may serve as more accurate surrogates for conventional measurements of body composition. A novel and excellent model incorporating the SMI and radiomics features showed favorable power for predicting grade 3/4 gastrointestinal toxicity. Such quantitative predictive models may potentially be useful for precision medicine and clinical treatment decisions in LARC patients. Further external validation in larger samples is needed in subsequent studies for clinical application.

## CRediT authorship contribution statement

**Wang Yang:** Conceptualization, Methodology, Data curation. **Zhiyuan Zhang:** Conceptualization, Methodology, Data curation. **Menglong Zhou:** Data curation. **Jiazhou Wang:** Methodology. **Yan Wang:** Data curation. **Fan Xia:** Conceptualization, Methodology, Supervision. **Zhen Zhang:** Supervision.

## Funding

This work was funded by the National Natural Science Foundation of China (Grant No. 81773357), Shanghai Anticancer Association EYAS PROJECT (Grant Nos. SACA-CY20C14, SACA-CY20B07) and Cancer Precision Radiotherapy Spark Program of China International Medical Foundation (Grant No. 2019-N-11).

## Declaration of Competing Interest

The authors declare that they have no known competing financial interests or personal relationships that could have appeared to influence the work reported in this paper.

## References

[1] Siegel RL, Miller KD, Jemal A. Cancer statistics, 2020. CA: a cancer journal for clinicians. 2020 1(70):7-30.10.3322/caac.21590. 10.3322/caac.21590 31912902

[2] Bosset J.F., Calais G., Mineur L., Maingon P., Radosevic-Jelic L., Daban A. (2005). Enhanced tumorocidal effect of chemotherapy with preoperative radiotherapy for rectal cancer: preliminary results–EORTC 22921. J CLIN ONCOL.

[3] Bosset J.F., Collette L., Calais G., Mineur L., Maingon P., Radosevic-Jelic L. (2006). Chemotherapy with preoperative radiotherapy in rectal cancer. N Engl J Med.

[4] Sauer R., Liersch T., Merkel S., Fietkau R., Hohenberger W., Hess C. (2012). Preoperative Versus Postoperative Chemoradiotherapy for Locally Advanced Rectal Cancer: Results of the German CAO/ARO/AIO-94 Randomized Phase III Trial After a Median Follow-Up of 11 Years. J CLIN ONCOL.

[5] Wong S.J., Winter K., Meropol N.J., Anne P.R., Kachnic L., Rashid A. (2012). Radiation Therapy Oncology Group 0247: a randomized Phase II study of neoadjuvant capecitabine and irinotecan or capecitabine and oxaliplatin with concurrent radiotherapy for patients with locally advanced rectal cancer. Int J Radiat Oncol Biol Phys.

[6] Hong Y.S., Kim D.Y., Lim S.B., Choi H.S., Jeong S.Y., Jeong J.Y. (2011). Preoperative chemoradiation with irinotecan and capecitabine in patients with locally advanced resectable rectal cancer: long-term results of a Phase II study. Int J Radiat Oncol Biol Phys.

[7] Zhu J., Liu A., Sun X., Liu L., Zhu Y., Zhang T. (2020). Multicenter, Randomized, Phase III Trial of Neoadjuvant Chemoradiation With Capecitabine and Irinotecan Guided by UGT1A1 Status in Patients With Locally Advanced Rectal Cancer. J CLIN ONCOL.

[8] Willeke F., Horisberger K., Kraus-Tiefenbacher U., Wenz F., Leitner A., Hochhaus A. (2007). A phase II study of capecitabine and irinotecan in combination with concurrent pelvic radiotherapy (CapIri-RT) as neoadjuvant treatment of locally advanced rectal cancer. Br J Cancer.

[9] Prado C.M., Lieffers J.R., McCargar L.J., Reiman T., Sawyer M.B., Martin L. (2008). Prevalence and clinical implications of sarcopenic obesity in patients with solid tumours of the respiratory and gastrointestinal tracts: a population-based study. LANCET ONCOL.

[10] Chung E., Lee H.S., Cho E.S., Park E.J., Baik S.H., Lee K.Y. (2020). Prognostic significance of sarcopenia and skeletal muscle mass change during preoperative chemoradiotherapy in locally advanced rectal cancer. CLIN NUTR.

[11] Choi M.H., Oh S.N., Lee I.K., Oh S.T., Won D.D. (2018). Sarcopenia is negatively associated with long-term outcomes in locally advanced rectal cancer. J Cachexia Sarcopenia Muscle.

[12] Bedrikovetski S., Traeger L., Price T.J., Carruthers S., Selva-Nayagam S., Moore J.W. (2023). Can sarcopenia predict complete response after total neoadjuvant therapy in advanced rectal cancer? A multicentre observational cohort study. J SURG ONCOL.

[13] Giani A, Famularo S, Riva L, Tamini N, Ippolito D, Nespoli L, et al. Association between specific presurgical anthropometric indexes and morbidity in patients undergoing rectal cancer resection. NUTRITION. 202075-76):110779.10.1016/j.nut.2020.110779. 10.1016/j.nut.2020.110779 32268263

[14] Li Q., An T., Wu J., Lu W., Wang Y., Li J. (2023). The impact of sarcopenia on the outcome of patients with left-sided colon and rectal cancer after curative surgery. BMC Cancer.

[15] Drami I., Pring E.T., Gould L., Malietzis G., Naghibi M., Athanasiou T. (2021). Body Composition and Dose-limiting Toxicity in Colorectal Cancer Chemotherapy Treatment; a Systematic Review of the Literature. Could Muscle Mass be the New Body Surface Area in Chemotherapy Dosing?. Clin Oncol (r Coll Radiol).

[16] Sealy M.J., Dechaphunkul T., van der Schans C.P., Krijnen W.P., Roodenburg J., Walker J. (2020). Low muscle mass is associated with early termination of chemotherapy related to toxicity in patients with head and neck cancer. CLIN NUTR.

[17] Barret M., Antoun S., Dalban C., Malka D., Mansourbakht T., Zaanan A. (2014). Sarcopenia is linked to treatment toxicity in patients with metastatic colorectal cancer. NUTR CANCER.

[18] Chen L.K., Liu L.K., Woo J., Assantachai P., Auyeung T.W., Bahyah K.S. (2014). Sarcopenia in Asia: consensus report of the Asian Working Group for Sarcopenia. J AM MED DIR ASSOC.

[19] Cruz-Jentoft A.J., Bahat G., Bauer J., Boirie Y., Bruyère O., Cederholm T. (2019). Sarcopenia: revised European consensus on definition and diagnosis. AGE AGEING.

[20] Aerts HJ, Velazquez ER, Leijenaar RT, Parmar C, Grossmann P, Carvalho S, et al. Decoding tumour phenotype by noninvasive imaging using a quantitative radiomics approach. NAT COMMUN. 20145):4006.10.1038/ncomms5006. 10.1038/ncomms5006 PMC4059926 24892406

[21] Weng Q., Zhou L., Wang H., Hui J., Chen M., Pang P. (2019). A radiomics model for determining the invasiveness of solitary pulmonary nodules that manifest as part-solid nodules. CLIN RADIOL.

[22] Cai J., Zheng J., Shen J., Yuan Z., Xie M., Gao M. (2020). A Radiomics Model for Predicting the Response to Bevacizumab in Brain Necrosis after Radiotherapy. CLIN CANCER RES.

[23] Lin P., Yang P.F., Chen S., Shao Y.Y., Xu L., Wu Y. (2020). A Delta-radiomics model for preoperative evaluation of Neoadjuvant chemotherapy response in high-grade osteosarcoma. Cancer Imaging.

[24] Biason P., Masier S., Toffoli G. (2008). UGT1A1*28 and other UGT1A polymorphisms as determinants of irinotecan toxicity. J Chemother.

[25] (2017). Version 50.

[26] Yang W., Xia F., Wang J., Zhou M., Li G., Shen L. (2020). Quantifying skeletal muscle wasting during chemoradiotherapy with Jacobian calculations for the prediction of survival and toxicity in patients with gastric cancer. Eur J Surg Oncol.

[27] Tibshirani R. (1997). The lasso method for variable selection in the Cox model. STAT MED.

[28] Poldrack R.A., Huckins G., Varoquaux G. (2020). Establishment of Best Practices for Evidence for Prediction: A Review. JAMA PSYCHIAT.

[29] Vickers A.J., Elkin E.B. (2006). Decision curve analysis: a novel method for evaluating prediction models. MED DECIS MAKING.

[30] Vickers AJ, Van Calster B, Steyerberg EW. Net benefit approaches to the evaluation of prediction models, molecular markers, and diagnostic tests. BMJ. 2016352):i6.10.1136/bmj.i6. 10.1136/bmj.i6 PMC4724785 26810254

[31] Fitzgerald M., Saville B.R., Lewis R.J. (2015). Decision curve analysis. JAMA.

[32] Kerr K.F., Brown M.D., Zhu K., Janes H. (2016). Assessing the Clinical Impact of Risk Prediction Models With Decision Curves: Guidance for Correct Interpretation and Appropriate Use. J CLIN ONCOL.

[33] Collins G.S., Reitsma J.B., Altman D.G., Moons K. (2015). Transparent Reporting of a Multivariable Prediction Model for Individual Prognosis or Diagnosis (TRIPOD): The TRIPOD Statement. EUR UROL.

[34] Prado C.M., Baracos V.E., McCargar L.J., Mourtzakis M., Mulder K.E., Reiman T. (2007). Body Composition as an Independent Determinant of 5-Fluorouracil-Based Chemotherapy Toxicity. CLIN CANCER RES.

[35] Tan B.H., Brammer K., Randhawa N., Welch N.T., Parsons S.L., James E.J. (2015). Sarcopenia is associated with toxicity in patients undergoing neo-adjuvant chemotherapy for oesophago-gastric cancer. Eur J Surg Oncol.

[36] Prado C.M., Baracos V.E., McCargar L.J., Reiman T., Mourtzakis M., Tonkin K. (2009). Sarcopenia as a determinant of chemotherapy toxicity and time to tumor progression in metastatic breast cancer patients receiving capecitabine treatment. CLIN CANCER RES.

[37] Antoun S., Baracos V.E., Birdsell L., Escudier B., Sawyer M.B. (2010). Low body mass index and sarcopenia associated with dose-limiting toxicity of sorafenib in patients with renal cell carcinoma. ANN ONCOL.

[38] Bousabarah K., Temming S., Hoevels M., Borggrefe J., Baus W.W., Ruess D. (2019). Radiomic analysis of planning computed tomograms for predicting radiation-induced lung injury and outcome in lung cancer patients treated with robotic stereotactic body radiation therapy. STRAHLENTHER ONKOL.

[39] Abdollahi H., Mahdavi S.R., Mofid B., Bakhshandeh M., Razzaghdoust A., Saadipoor A. (2018). Rectal wall MRI radiomics in prostate cancer patients: prediction of and correlation with early rectal toxicity. INT J RADIAT BIOL.

[40] de Jong E, Sanders K, Deist TM, van Elmpt W, Jochems A, van Timmeren JE, et al. Can radiomics help to predict skeletal muscle response to chemotherapy in stage IV non-small cell lung cancer? EUR J CANCER. 2019120):107-13.10.1016/j.ejca.2019.07.023. 10.1016/j.ejca.2019.07.023 31514107

[41] Akay M. (1995). Wavelets in biomedical engineering. ANN BIOMED ENG.

[42] Mai D., Drami I., Pring E.T., Gould L.E., Lung P., Popuri K. (2023). A systematic review of automated segmentation of 3D computed-tomography scans for volumetric body composition analysis. J Cachexia Sarcopenia Muscle.

