# Supplementary material for: Radiomics of skeletal muscle helps to predict gastrointestinal toxicity in locally advanced rectal cancer patients receiving neoadjuvant chemoradiotherapy
Source: Clin Transl Radiat Oncol. 2023 Nov 20;44:100703. doi: 10.1016/j.ctro.2023.100703 (PMC10701125; doi:10.1016/j.ctro.2023.100703)

**Supplementary Material**

Table E1. Extracted radiomics features.

| Group | Radiomics Features |
| --- | --- |
| Shape and size-based features | Volume  Area  Volume to area ratio |
| First order statistical features | Mean_Intensity  Median_Intensity  Variance_Intensity  Skewness_Intensity  Kurtosis_Intensity  Minimum_Intensity  Maximum_Intensity  Std_Intensity  Range_Intensity  Scaled_mean  Scaled_median  Scaled_variance  Scaled_skewness  Scaled_kurtosis  Scaled_entropy  Scaled_min  Scaled_max  Scaled_std  Scaled_range |
| Textural features | GLCM_Contrast  GLCM_Correlation  GLCM_Energy  GLCM_Homogeneity  GLCM_Mean  GLCM_Variance  GLCM_Std  GLCM_Dissimilarity  GLCM_Entropy  GLCM_Sum_average  GLCM_Difference_average  GLCM_Sum_variance  GLCM_Difference_variance  GLCM_Sum_Entropy  GLCM_Difference_Entropy  GLCM_Information_Measures_I  GLCM_Information_Measures_II  GLCM_Maximal_Correlation_Coefficient  GLCM_Homogeneity_Original  GLCM_Correlation_Original  GLRLM_SRE  GLRLM_LRE  GLRLM_GLN  GLRLM_RLN  GLRLM_RP  GLRLM_LGRE  GLRLM_HGRE  GLRLM_SRLGE  GLRLM_SRHGE  GLRLM_LRLGE  GLRLM_LRHGE |
| Wavelet features | Wavelet(LL)_GLCM_Contrast  Wavelet(LL)_GLCM_Correlation  Wavelet(LL)_GLCM_Energy  Wavelet(LL)_GLCM_Homogeneity  Wavelet(LL)_GLCM_Mean  Wavelet(LL)_GLCM_Variance  Wavelet(LL)_GLCM_Std  Wavelet(LL)_GLCM_Dissimilarity  Wavelet(LL)_GLCM_Entropy  Wavelet(LL)_GLCM_Sum_average  Wavelet(LL)_GLCM_Difference_average  Wavelet(LL)_GLCM_Sum_variance  Wavelet(LL)_GLCM_Difference_variance  Wavelet(LL)_GLCM_Sum_Entropy  Wavelet(LL)_GLCM_Difference_Entropy  Wavelet(LL)_GLCM_Information_Measures_I  Wavelet(LL)_GLCM_Information_Measures_II  Wavelet(LL)_GLCM_Correlation  Wavelet(LL)_GLCM_Homogeneity_Original  Wavelet(LL)_GLCM_Correlation_Original  Wavelet(LH)_GLCM_Contrast  Wavelet(LH)_GLCM_Correlation  Wavelet(LH)_GLCM_Energy  Wavelet(LH)_GLCM_Homogeneity  Wavelet(LH)_GLCM_Mean  Wavelet(LH)_GLCM_Variance  Wavelet(LH)_GLCM_Std  Wavelet(LH)_GLCM_Dissimilarity  Wavelet(LH)_GLCM_Entropy  Wavelet(LH)_GLCM_Sum_average  Wavelet(LH)_GLCM_Difference_average  Wavelet(LH)_GLCM_Sum_variance  Wavelet(LH)_GLCM_Difference_variance  Wavelet(LH)_GLCM_Sum_Entropy  Wavelet(LH)_GLCM_Difference_Entropy  Wavelet(LH)_GLCM_Information_Measures_I  Wavelet(LH)_GLCM_Information_Measures_II  Wavelet(LH)_GLCM_Maximal_Correlation_Coefficient  Wavelet(LH)_GLCM_Homogeneity_Original  Wavelet(LH)_GLCM_Correlation_Original  Wavelet(HL)_GLCM_Contrast  Wavelet(HL)_GLCM_Correlation  Wavelet(HL)_GLCM_Energy  Wavelet(HL)_GLCM_Homogeneity  Wavelet(HL)_GLCM_Mean  Wavelet(HL)_GLCM_Variance  Wavelet(HL)_GLCM_Std  Wavelet(HL)_GLCM_Dissimilarity  Wavelet(HL)_GLCM_Entropy  Wavelet(HL)_GLCM_Sum_average  Wavelet(HL)_GLCM_Difference_average  Wavelet(HL)_GLCM_Sum_variance  Wavelet(HL)_GLCM_Difference_variance  Wavelet(HL)_GLCM_Sum_Entropy  Wavelet(HL)_GLCM_Difference_Entropy  Wavelet(HL)_GLCM_Information_Measures_I  Wavelet(HL)_GLCM_Information_Measures_II  Wavelet(HL)_GLCM_Maximal_Correlation_Coefficient  Wavelet(HL)_GLCM_Homogeneity_Original  Wavelet(HL)_GLCM_Correlation_Original  Wavelet(HH)_GLCM_Contrast  Wavelet(HH)_GLCM_Correlation  Wavelet(HH)_GLCM_Energy  Wavelet(HH)_GLCM_Homogeneity  Wavelet(HH)_GLCM_Mean  Wavelet(HH)_GLCM_Variance  Wavelet(HH)_GLCM_Std  Wavelet(HH)_GLCM_Dissimilarity  Wavelet(HH)_GLCM_Entropy  Wavelet(HH)_GLCM_Sum_average  Wavelet(HH)_GLCM_Difference_average  Wavelet(HH)_GLCM_Sum_variance  Wavelet(HH)_GLCM_Difference_variance  Wavelet(HH)_GLCM_Sum_Entropy  Wavelet(HH)_GLCM_Difference_Entropy  Wavelet(HH)_GLCM_Information_Measures_I  Wavelet(HH)_GLCM_Information_Measures_II  Wavelet(HH)_GLCM_Maximal_Correlation_Coefficient  Wavelet(HH)_GLCM_Homogeneity_Original  Wavelet(HH)_GLCM_Correlation_Original  Wavelet(LL)_GLRLM_SRE  Wavelet(LL)_GLRLM_LRE  Wavelet(LL)_GLRLM_GLN  Wavelet(LL)_GLRLM_RLN  Wavelet(LL)_GLRLM_RP  Wavelet(LL)_GLRLM_LGRE  Wavelet(LL)_GLRLM_HGRE  Wavelet(LL)_GLRLM_SRLGE  Wavelet(LL)_GLRLM_SRHGE  Wavelet(LL)_GLRLM_LRLGE  Wavelet(LL)_GLRLM_LRHGE  Wavelet(LH)_GLRLM_SRE  Wavelet(LH)_GLRLM_LRE  Wavelet(LH)_GLRLM_GLN  Wavelet(LH)_GLRLM_RLN  Wavelet(LH)_GLRLM_RP  Wavelet(LH)_GLRLM_LGRE  Wavelet(LH)_GLRLM_HGRE  Wavelet(LH)_GLRLM_SRLGE  Wavelet(LH)_GLRLM_SRHGE  Wavelet(LH)_GLRLM_LRLGE  Wavelet(LH)_GLRLM_LRHGE  Wavelet(HL)_GLRLM_SRE  Wavelet(HL)_GLRLM_LRE  Wavelet(HL)_GLRLM_GLN  Wavelet(HL)_GLRLM_RLN  Wavelet(HL)_GLRLM_RP  Wavelet(HL)_GLRLM_LGRE  Wavelet(HL)_GLRLM_HGRE  Wavelet(HL)_GLRLM_SRLGE  Wavelet(HL)_GLRLM_SRHGE  Wavelet(HL)_GLRLM_LRLGE  Wavelet(HL)_GLRLM_LRHGE  Wavelet(HH)_GLRLM_SRE  Wavelet(HH)_GLRLM_LRE  Wavelet(HH)_GLRLM_GLN  Wavelet(HH)_GLRLM_RLN  Wavelet(HH)_GLRLM_RP  Wavelet(HH)_GLRLM_LGRE  Wavelet(HH)_GLRLM_HGRE  Wavelet(HH)_GLRLM_SRLGE  Wavelet(HH)_GLRLM_SRHGE  Wavelet(HH)_GLRLM_LRLGE  Wavelet(HH)_GLRLM_LRHGE  Wavelet(LL)_Mean_Intensity  Wavelet(LL)_Median_Intensity  Wavelet(LL)_Variance_Intensity  Wavelet(LL)_Skewness_Intesity  Wavelet(LL)_Kurtosis_Intensity  Wavelet(LL)_Minimum_Intensity  Wavelet(LL)_Maximum_Intensity  Wavelet(LL)_Std_Intensity  Wavelet(LL)_Range_Intensity  Wavelet(LL)_Scaled_mean  Wavelet(LL)_Scaled_median  Wavelet(LL)_Scaled_variance  Wavelet(LL)_Scaled_skewness  Wavelet(LL)_Scaled_kurtosis  Wavelet(LL)_Scaled_entropy  Wavelet(LL)_Scaled_min  Wavelet(LL)_Scaled_max  Wavelet(LL)_Scaled_std  Wavelet(LL)_Scaled_range  Wavelet(LH)_Mean_Intensity  Wavelet(LH)_Median_Intensity  Wavelet(LH)_Variance_Intensity  Wavelet(LH)_Skewness_Intensity  Wavelet(LH)_Kurtosis_Intensity  Wavelet(LH)_Minimum_Intensity  Wavelet(LH)_Maximum_Intensity  Wavelet(LH)_Std_Intensity  Wavelet(LH)_Range_Intensity  Wavelet(LH)_Scaled_mean  Wavelet(LH)_Scaled_median  Wavelet(LH)_Scaled_variance  Wavelet(LH)_Scaled_skewness  Wavelet(LH)_Scaled_kurtosis  Wavelet(LH)_Scaled_entropy  Wavelet(LH)_Scaled_min  Wavelet(LH)_Scaled_max  Wavelet(LH)_Scaled_std  Wavelet(LH)_Scaled_range  Wavelet(HL)_Mean_Intensity  Wavelet(HL)_Median_Intensity  Wavelet(HL)_Variance_Intensity  Wavelet(HL)_Skewness_Intensity  Wavelet(HL)_Kurtosis_Intensity  Wavelet(HL)_Minimun_Intensity  Wavelet(HL)_Maximum_Intensity  Wavelet(HL)_Std_Intensity  Wavelet(HL)_Range_Intensity  Wavelet(HL)_Scaled_mean  Wavelet(HL)_Scaled_median  Wavelet(HL)_Scaled_variance  Wavelet(HL)_Scaled_skewness  Wavelet(HL)_Scaled_kurtosis  Wavelet(HL)_Scaled_entropy  Wavelet(HL)_Scaled_min  Wavelet(HL)_Scaled_max  Wavelet(HL)_Scaled_std  Wavelet(HL)_Scaled_range  Wavelet(HH)_Mean_Intensity  Wavelet(HH)_Median_Intensity  Wavelet(HH)_Variance_Intensity  Wavelet(HH)_Skewness_Intensity  Wavelet(HH)_Kurtosis_Intensity  Wavelet(HH)_Minimum_Intensity  Wavelet(HH)_Maximum_Intensity  Wavelet(HH)_Std_Intensity  Wavelet(HH)_Range_Intensity  Wavelet(HH)_Scaled_mean  Wavelet(HH)_Scaled_median  Wavelet(HH)_Scaled_variance  Wavelet(HH)_Scaled_skewness  Wavelet(HH)_Scaled_kurtosis  Wavelet(HH)_Scaled_entropy  Wavelet(HH)_Scaled_min  Wavelet(HH)_Scaled_max  Wavelet(HH)_Scaled_std  Wavelet(HH)_Scaled_range |

**Abbreviations:** GLCM, Gray Level Co-occurrence Matrix; GLRLM, Gray Level Run Length Matrix; SRE, Short Run Emphasis; LRE, Long Run Emphasis; GLN, Gray-Level Nonuniformity; RLN, Run-Length Nonuniformity; RP, Run Percentage; LGRE, Low Gray-Level Run Emphasis; HGRE, High Gray-Level Run Emphasis; SRLGE, Short Run Low Gray-Level Emphasis; SRHGE, Short Run High Gray-Level Emphasis; LRLGE, Long Run Low Gray-Level Emphasis; LRHGE, Long Run High Gray-Level Emphasis

Table E2. Incidence and maximum toxicity observed during concurrent chemoradiotherapy (n=214 patients).

| Toxicities | Grade 1 | Grade 2 | Grade 3 | Grade 4 |
| --- | --- | --- | --- | --- |
| Overall toxicities | 23 (10.7%) | 82 (38.3%) | 86 (40.2%) | 20 (9.3%) |
| Gastrointestinal tract | 64 (29.9) | 87 (40.7%) | 58 (27.1%) | 6 (2.8%) |
| Diarrhea/ proctitis | 69 (32.2%) | 73 (34.1%) | 48 (22.4%) | 6 (2.8%) |
| Nausea/vomiting | 79 (36.9%) | 52 (24.3%) | 31 (14.5%) | - |
| Hematotoxicity | 38 (17.8%) | 81 (37.9%) | 52 (24.3%) | 14 (6.5%) |
| Leucopenia | 35 (16.4) | 81 (37.9%) | 54 (25.2%) | 7 (3.3%) |
| Neutropenia | 40 (18.7%) | 49 (22.9%) | 33 (15.4%) | 13 (6.1%) |
| Thrombocytopenia | 15 (7.0%) | 3 (1.4%) | 2 (0.9%) | 1 (0.5%) |
| Anemia | 87 (40.7%) | 29 (13.6%) | 2 (0.9%) | 0 (0.0%) |

Table E3. Coefficients of the nine selected features.

| Feature selected | Coefficient |
| --- | --- |
| (Intercept) | -1.16692675 |
| SMI | -0.67645259 |
| Wavelet(LL)_GLCM_Correlation | -0.20272008 |
| Wavelet(LL)_GLRLM_SRLGE | -0.09908598 |
| Wavelet(HL)_GLRLM_SRE | -0.03477819 |
| Wavelet(LH)_Maximum_Intensity | 0.01039565 |
| Wavelet(HH)_Median_Intensity | 0.02054542 |
| Maximum_Intensity | 0.04699865 |
| Wavelet(LH)_Mean_Intensity | 0.16058016 |
| Wavelet(LL)_GLCM_Information_Measures_II | 0.28162851 |

Abbreviations: SMI, skeletal muscle index; GLCM, Grey Level Co-occurrence Matrix; GLRLM, Grey Level Run Length Matrix; SRLGE, Short Run Low Grey-Level Emphasis; SRE, Short Run Emphasis

Table E4. Predictive efficacy of the two predictive models.

|  | Training dataset (n = 115) | | Internal validation dataset (n = 49) | | External validation dataset (n = 50) | |
| --- | --- | --- | --- | --- | --- | --- |
|  | SMI | SMI+radiomics | SMI | SMI+radiomics | SMI | SMI+radiomics |
| Sensitivity, % | 55.2% | 86.2% | 66.7% | 80.0% |  | 70.6% |
| Specificity, % | 82.6% | 68.6% | 76.5% | 79.4% |  |  |
| Accuracy, % | 75.7% | 73.0% | 73.5% | 79.6% |  |  |
| Precision, % |  | 48.1% |  | 63.2% |  | 63.2% |
| AUC (95% CI) | 0.739 (0.639-0.839) | 0.856 (0.782-0.929) | 0.741 (0.594-0.889) | 0.812 (0.667-0.956) | 0.741 (0.594-0.889) | 0.812 (0.667-0.956) |
| F1-score |  | 0.62 |  | 0.71 |  | 0.67 |

Abbreviations: SMI, skeletal muscle index; AUC, area under the curve; CI, confidence interval

Figure E1. Feature selection using the LASSO logistic regression model. (a) Tuning parameter (λ) selection in the LASSO model used 10-fold cross-validation via minimum criteria. The AUC was plotted as a function of log(λ). The dotted vertical lines were defined as the optimal λ by using the minimum criteria and the 1-standard error of the minimum criteria (the 1-SE criteria). A λ value of 0.04795 with log (λ)= -3.03767 was selected (1-SE criteria). (b) LASSO coefficient profiles of the 254 features. The dotted vertical line was plotted at the given λ. For the optimal λ, nine features with nonzero coefficients are indicated.

Abbreviations: LASSO, least absolute shrinkage and selection operator; AUC, area under the receiver operator characteristic curve


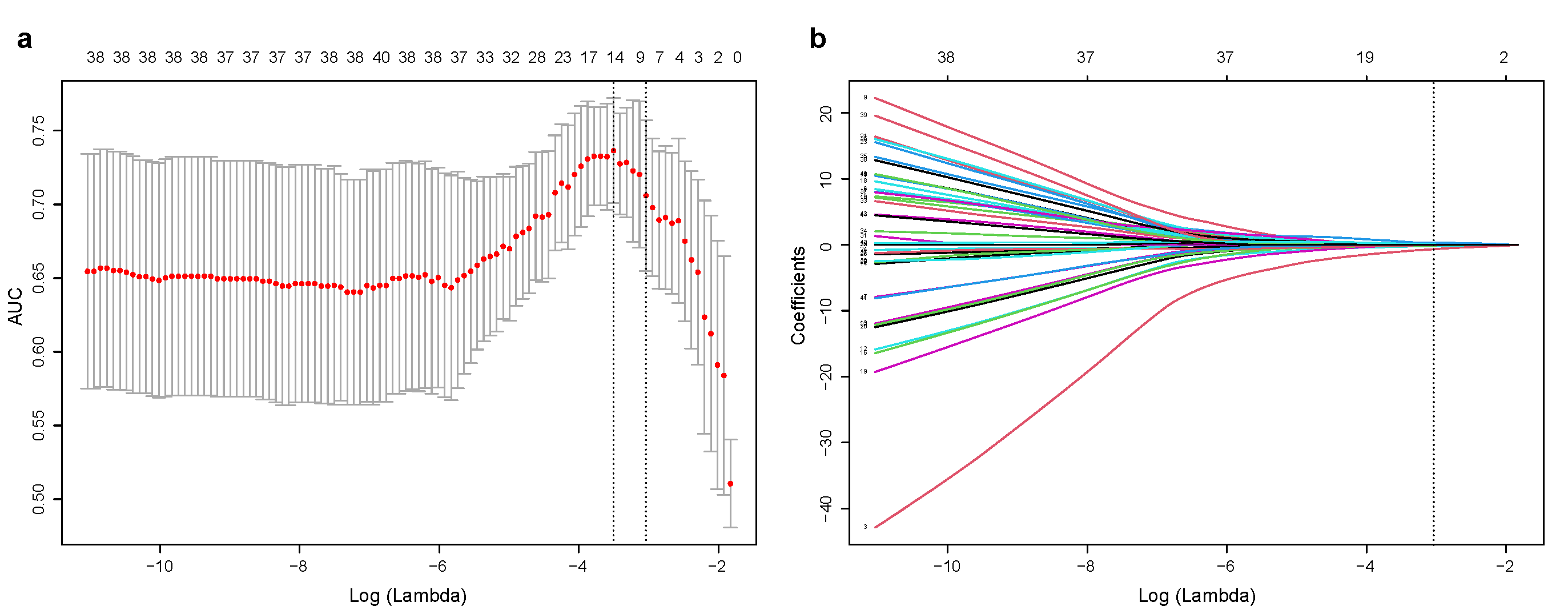

Supplement: Supplementary data 1 [file mmc1.docx]
